# Supplementary material for: Warming assessment of the bottom-up Paris Agreement emissions pledges
Source: Nat Commun. 2018 Nov 16;9:4810. doi: 10.1038/s41467-018-07223-9 (PMC6240108; doi:10.1038/s41467-018-07223-9)
Supplement: Supplementary file 1 — Supplementary Information [file 41467_2018_7223_MOESM1_ESM.pdf]

Supplementary Information

Warming assessment of the bottom-up Paris Agreement emissions pledges

Robiou du Pont, Y., Meinshausen, M.

This Supplementary Information provides figures and tables to support the Methods section of the study (section 1) and indicates how to read the Supplementary Data (section 2).

Contents

1. Supplementary Figures 3
2. Supplementary Tables 10
3. Supplementary Note 12
4. Supplementary References 13

1. Supplementary Figures

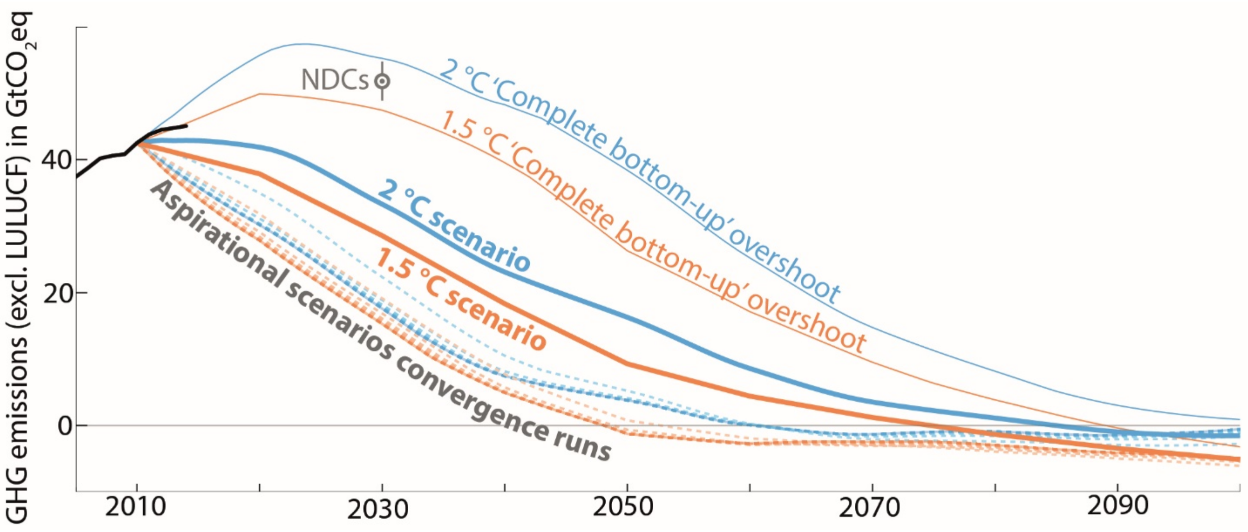

Supplementary Figure 1 | Scenarios towards 2 °C and 1.5 °C (thick solid blue and red lines) shown with their corresponding Complete bottom-up allocation (thin lines) and aspirational scenarios convergence runs (thin dashed lines). Unconditional, conditional and average (I)NDC assessment are shown in grey. LULUCF and bunker emissions are excluded. Converging aspirational scenarios (thin dotted lines) converge over 15 runs towards 2 °C and 1.5 °C (thin solid lines).

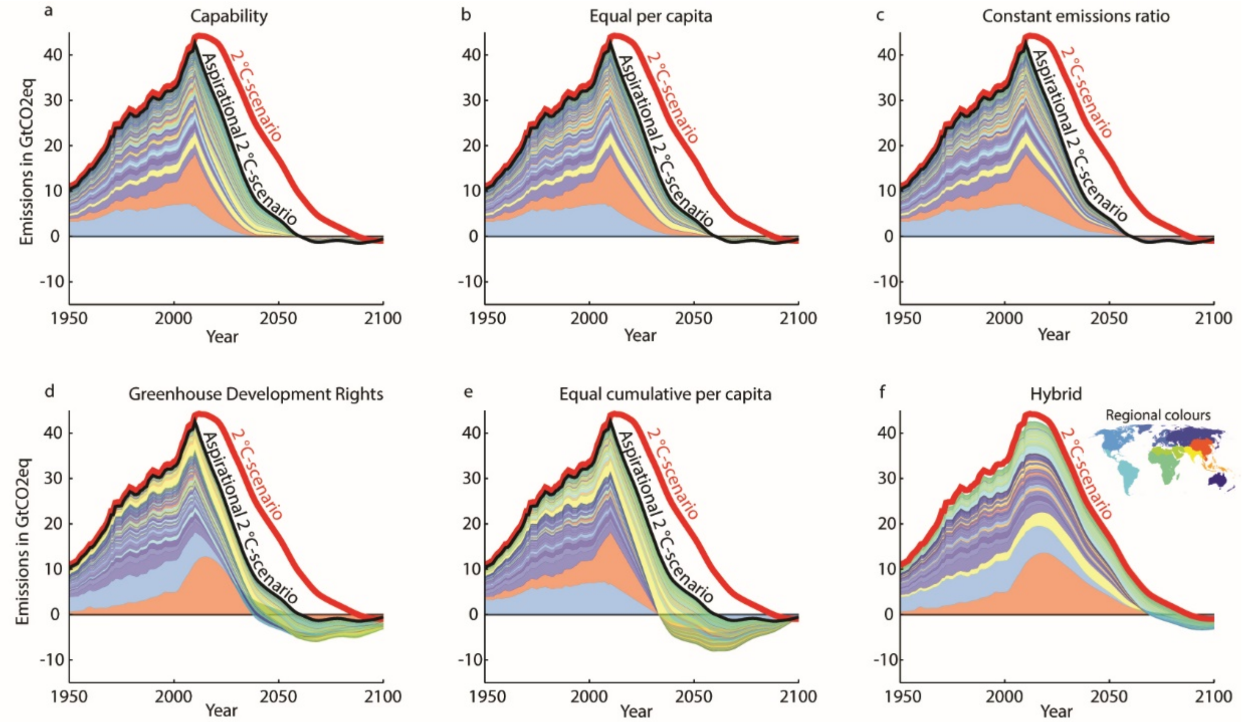

Supplementary Figure 2 | Distribution of the aspirational pathway towards 2 °C according to the five equity approaches and under a Complete bottom-up approach. The bottom-up distribution (based on largest cumulative emissions by 2100) of the aspirational 2 °C-scenario (black line) results in emissions matching that 2 °C-scenario (RCP2.6 excluding LULUCF emissions, red line). Each colour patch represents a country.

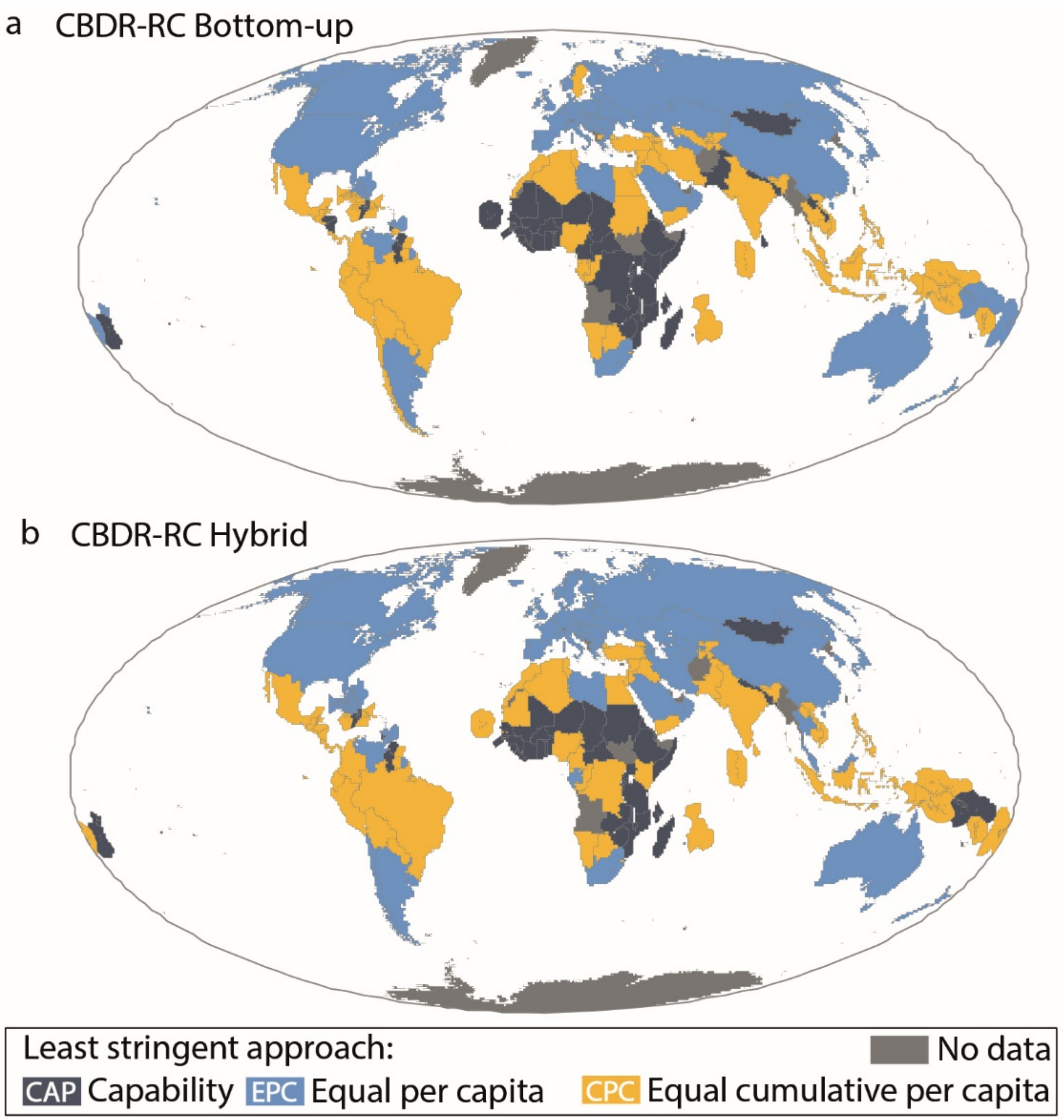

Supplementary Figure 3 | Least-stringent of three approaches (CAP, EPC and CPC), by lowest 2030 emissions, under a CBDR-RC bottom-up allocation (panel **a**), and CBDR-RC hybrid allocation (panel **b**) of the 2°C-scenario.

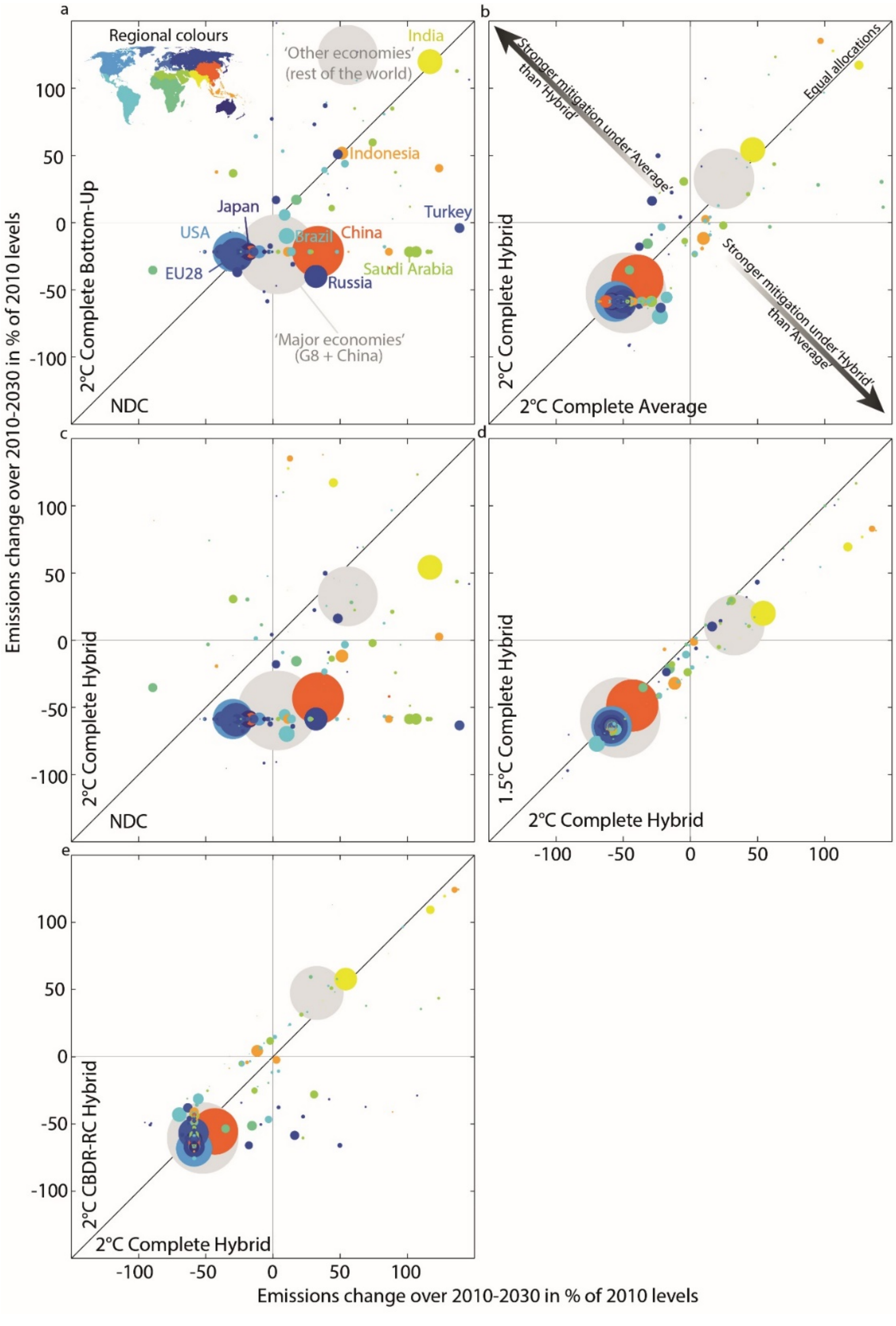

Supplementary Figure 4 | Comparison of emissions changes by 2030 under the Complete bottom-up, Complete hybrid, and CBDR-RC hybrid, the average of effort-sharing allocations and with (I)NDCs. a, Comparison of the Complete bottom-up (with the five effort-sharing approaches) allocation of the 2°C-scenario and countries NDCs ***b***, Comparison of the Complete hybrid allocation and the average of the five effort-sharing allocations, under the 2°C-scenario. **c**, Comparison of the Complete hybrid allocation of the 2°C-scenario and countries' NDCs. **d**, Comparison of the Complete hybrid allocation of the 2°C-scenario and 1.5°C-scenario. ***e***, Comparison of the CBDR-RC hybrid with the Complete hybrid. Disks' sizes are proportional to 2010 emissions level. Colours indicate countries' world regions, and the Major economies (G8+China, larger disk) and the Other economies (smaller disk) are shown in grey. The NDC evaluation follows the average evaluation of ref. [5].

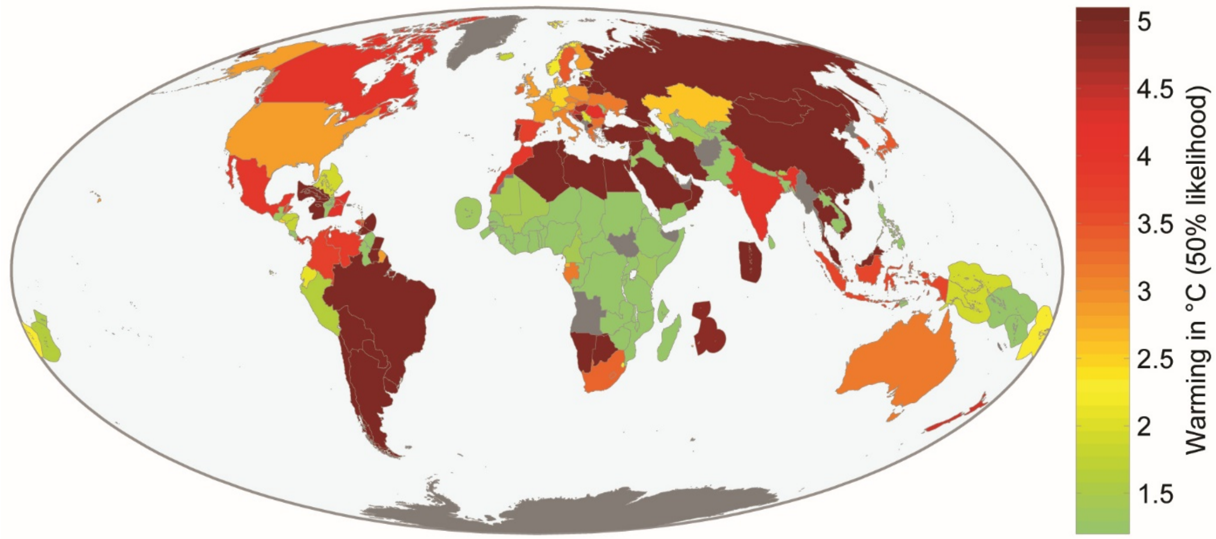

Supplementary Figure 5 | Global warming responses under a Complete hybrid approach following NDC ambitions without interpolation. Global warming responses (median assessment) following NDC ambitions.

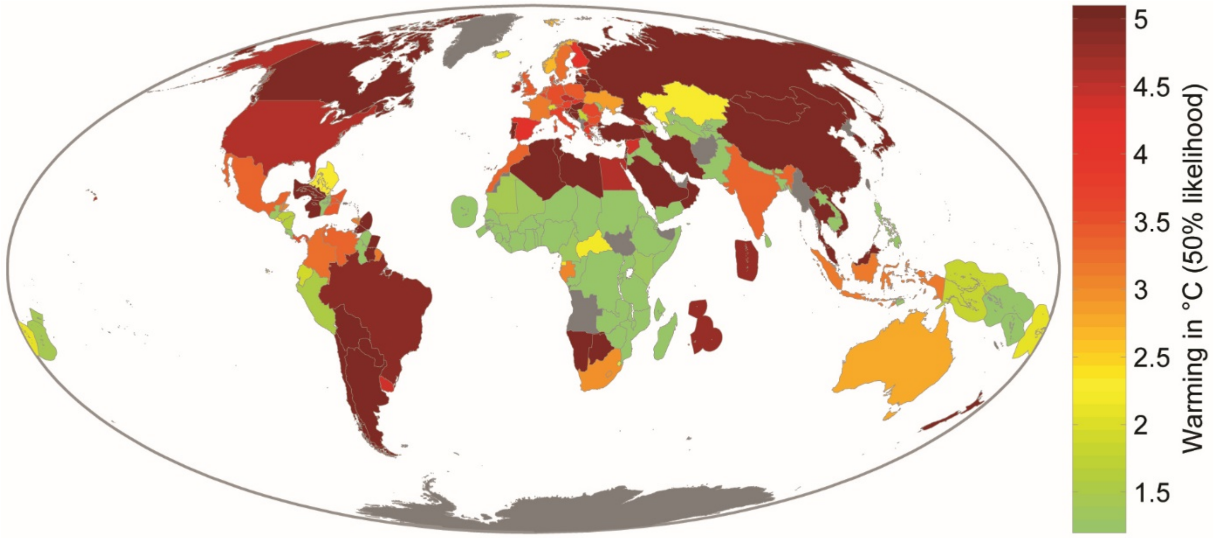

Supplementary Figure 6 | Global warming responses under a hybrid approach, including the GDR but excluding grandfathering, following NDC ambitions. Global warming responses (median assessment) following NDC ambitions.

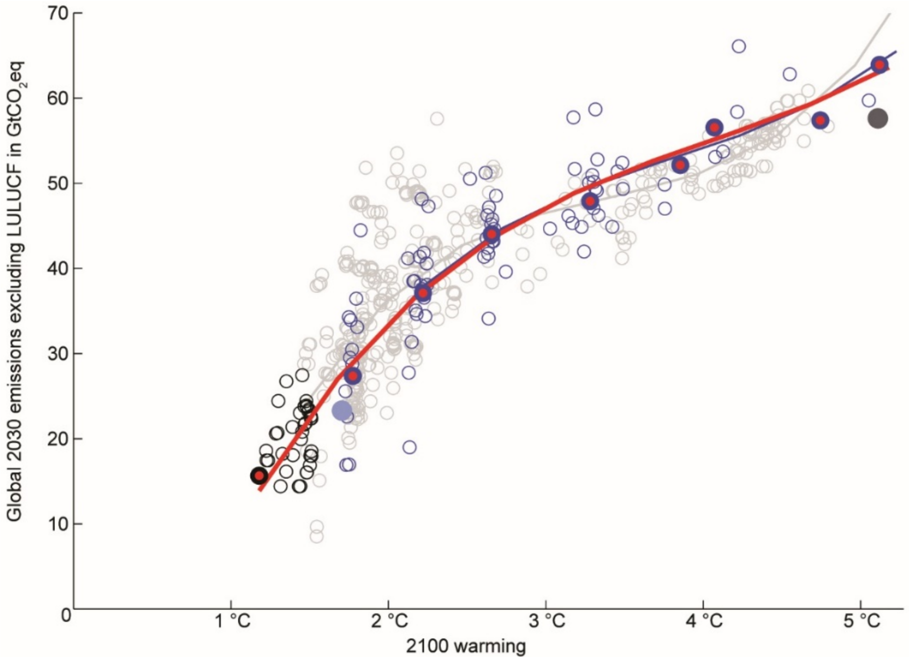

Supplementary Figure 7 | Selected global scenarios' 2030 emissions levels, excluding LULUCF, as a function of 2100 global warming. The 9 scenarios are selected (red filling) amongst the 85 scenarios from the SSP-database (blue circles) and the 36 scenarios from ref. [1] (black circles) to align with the third-degree polynomial fit (red line). The 412 IPCC-AR5 scenarios with available data (grew circles) are shown with their third-degree polynomial fit (grey line). The 1.5 °C-scenario, the 2 °C-scenario (RCP2.6, blue disk) and the business-as-usual scenario (RCP8.5, grey disk) are shown for comparison.

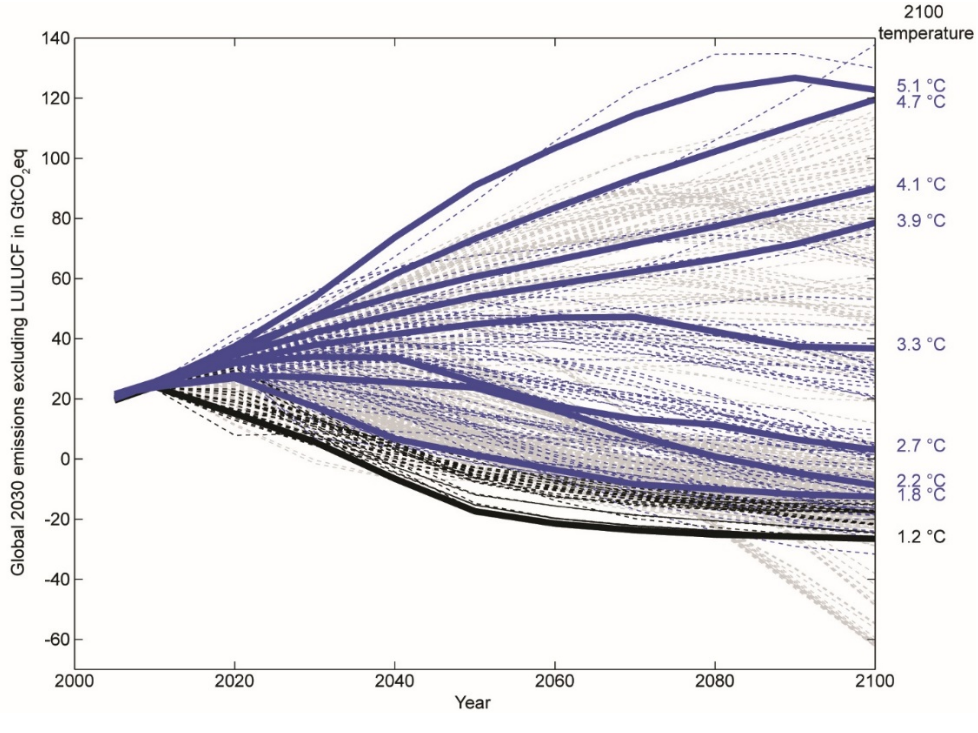

Supplementary Figure 8 | Selected global emissions scenarios. The scenario sub-selection (thick lines) from the 85 scenarios amongst the SSP-database (dashed blue lines) and the 36 scenarios from ref. [1] (dashed black lines) are shown with their 2100 warming assessments. The 412 IPCC-AR5 scenarios with available data are shown for comparison (grey dashed line).

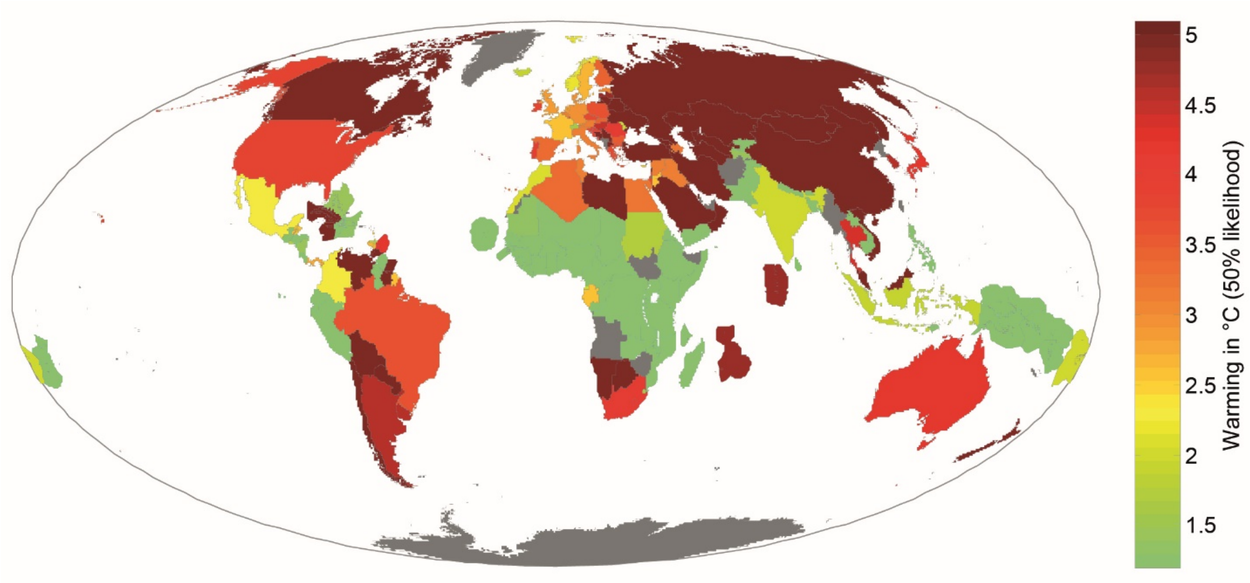

Supplementary Figure 9 | Global warming responses under a CBDR-RC hybrid approach, following high quantifications of NDC[5]. Global warming assessment (50% likelihood, compared to pre-industrial levels) of high NDC quantifications for 169 countries, as calculated in Figure 3a using the quadratic curve fit. The assessment ranges from 1.2°C to 5.1°C, NDCs outside this range are not differentiated. Small island developing states are represented by their maritime zones.

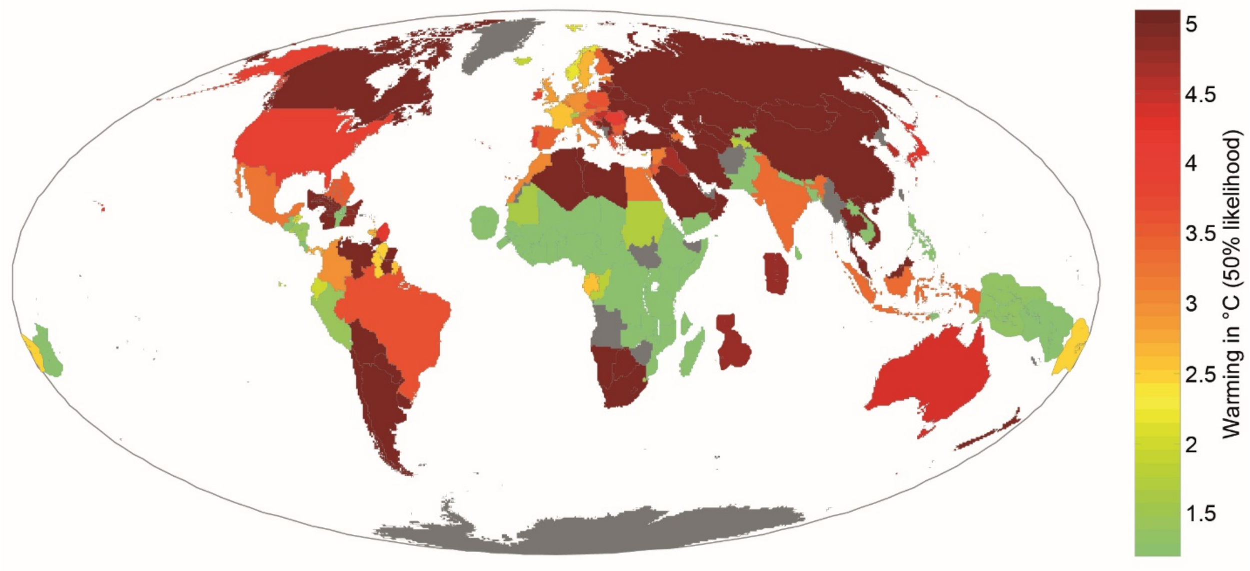

Supplementary Figure 10 | Global warming responses under a CBDR-RC hybrid approach, following low quantifications of NDC accounting for conditional pledges[5]. Global warming assessment (50% likelihood, compared to pre-industrial levels) of low NDC quantifications for 169 countries, as calculated in Figure 3a using the quadratic curve fit. The assessment ranges from 1.2°C to 5.1°C, NDCs outside this range are not differentiated. Small island developing states are represented by their maritime zones.

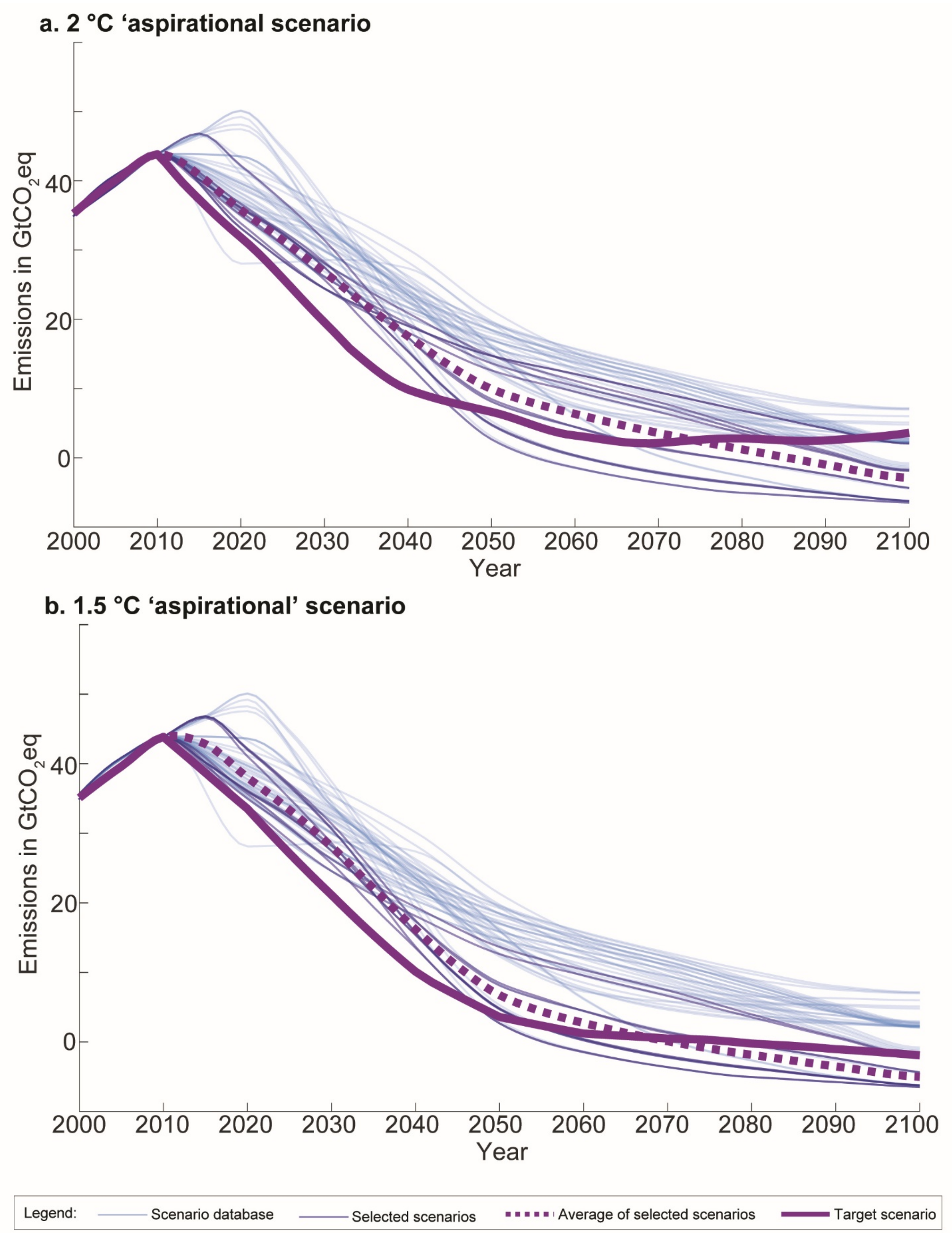

Supplementary Figure 11 | Scenario selection for the determination of the GHG composition, including bunker emissions, of the 1.5 °C aspirational scenarios (panels a.) and 2 °C aspirational (panel b.).

2. Supplementary Tables

Supplementary Table 1 | Description of the allocation setups.

Approach	Approach's setup	In Figure
CBDR-RC bottom-up	Each country follows the least-stringent effort-sharing allocation, that with the highest 2030 emissions, of a global emissions trajectory. As a result, this trajectory is overshot. Only three equity approaches are used EPC (for equality), CPC (for historical responsibility) and CAP approaches are used.	Figure 2
Complete bottom-up	Each country follows the least-stringent effort-sharing allocation, that with the highest cumulative emissions between 2010 and 2100, of a global emissions trajectory. As a result, this trajectory is overshot. All five effort-sharing approaches are used (including the grandfathering and GDR approaches).	Figure 1
CBDR-RC hybrid	Each country follows the least-stringent effort-sharing allocation, that with the highest 2030 emissions, of a virtual emissions trajectory lower than the targeted global emissions scenario. As a result, the targeted global emissions scenario is achieved. Only three equity approaches are used EPC (for equality), CPC (for historical responsibility) and CAP approaches are used.	Figure 2<br>Figure 3
Complete hybrid	Each country follows the least-stringent effort-sharing allocation, that with the highest cumulative emissions between 2010 and 2100, of a virtual emissions trajectory lower than the targeted global emissions scenario. As a result, the targeted global emissions scenario is achieved. All five effort-sharing approaches are used (including the grandfathering and GDR approaches).	Figure 1

Supplementary Table 2 | Equity approaches allocated to countries under the Complete bottom-up allocation of the 2°C-scenario used in Figure 1.

Least stringent approach	Country ISO ALPHA-3 codes
Capability: Countries with high GDP per capita have low emissions allocations.	35 countries: BDI, BEN, BFA, BGD, CIV, CMR, COM, DJI, ERI, ETH, GHA, GIN, GNB, GRD, HTI, LBR, LSO, MDG, MLI, MOZ, MWI, NER, NPL, RWA, SEN, SLE, SOM, STP, TCD, TGO, TON, TZA, UGA, ZMB, ZWE
Equal per Capita: Convergence towards equal annual emissions per person in 2040.	5 countries: BRB, CUB, LCA, VCT, WSM
Greenhouse Development Rights: Countries with high GDP per capita and high historical emissions per capita have low emissions allocation[2–4].	41 countries: ARM, AZE, BGR, BHR, BIH, BLR, BOL, CAF, COD, COG, CYP, DZA, GEO, GUY, HRV, HUN, IRQ, JOR, KAZ, KGZ, LVA, MDA, MKD, MLT, MNG, MRT, NAM, POL, PRY, ROU, RUS, SDN, SLB, SRB, TJK, TKM, UKR, UZB, VEN, YEM, ZAF
Equal cumulative per capita: Populations with high historical emissions have low emissions allocations.	44 countries: BLZ, BRA, BTN, BWA, COL, CPV, CRI, DOM, ECU, EGY, FJI, GMB, GNQ, GTM, HND, IDN, IND, JAM, KEN, KHM, LAO, LBN, LKA, MAR, MDV, MEX, MUS, NGA, NIC, PAK, PAN, PER, PHL, PNG, PRI, SLV, SUR, SWZ, SYR, TLS, TUN, TUR, VNM, VUT
Constant emissions ratios: Maintains current emissions ratios, preserves status-quo.	47 countries: ARG, AUS, AUT, BEL, BHS, BRN, CAN, CHE, CHL, CHN, CZE, DEU, DNK, ESP, EST, FIN, FRA, GAB, GBR, GRC, HKG, IRL, IRN, ISL, ISR, ITA, JPN, KOR, KWT, LBY, LTU, LUX, MYS, NLD, NOR, NZL, OMN, PRT, SAU, SGP, SVK, SVN, SWE47, THA, TTO, URY, USA

Here, the Complete bottom-up approach attributes to countries effort-sharing approaches that provide the greatest cumulative emissions between 2010 and 2100.

Supplementary Table 3 | Equity approaches attributed to countries under the CBDR-RC bottom-up allocation of the 2°C-scenario used in Figure 3.

Least stringent approach	Country ISO ALPHA-3 codes
Capability	48 countries: BDI, BEN, BFA, BGD, CAF, CIV, CMR, COD, COM, CPV, DJI, ERI, ETH, GHA, GIN, GMB, GNB, GRD, GUY, HND, HTI, KEN, LAO, LBR, LKA, LSO, MDG, MLI, MNG, MOZ, MRT, MWI, NER, NIC, NPL, PAK, RWA, SEN, SLE, SOM, STP, TCD, TGO, TON, TZA, UGA, ZMB, ZWE
Equal per Capita	61 countries: ARG, AUS, AUT, BEL, BGR, BHR, BHS, BIH, BLR, BRB, CAN, CHE, CHN, CYP, CZE, DEU, DNK, ESP, EST, FIN, FJI, FRA, GBR, GRC, HRV, HUN, IRL, ISL, ITA, JPN, KAZ, KOR, KWT, LBY, LCA, LTU, LUX, LVA, MDA, MLT, NLD, NOR, NZL, OMN, POL, PRT, ROU, RUS, SAU, SLB, SRB, SVK, SVN, TKM, UKR, URY, USA, VCT, VEN, WSM, ZAF
Equal cumulative per capita	63 countries: ARM, AZE, BLZ, BOL, BRA, BRN, BTN, BWA, CHL, COG, COL, CRI, CUB, DOM, DZA, ECU, EGY, GAB, GEO, GNQ, GTM, HKG, IDN, IND, IRN, IRQ, ISR, JAM, JOR, KGZ, KHM, LBN, MAR, MDV, MEX, MKD, MUS, MYS, NAM, NGA, PAN, PER, PHL, PNG, PRI, PRY, SDN, SGP, SLV, SUR, SWE, SWZ, SYR, THA, TJK, TLS, TTO, TUN, TUR, UZB, VNM, VUT, YEM

Here, the CBDR-RC bottom-up approach attributes to countries equity approaches that provide the highest 2030 emissions levels.

Supplementary Table 4 | Selected scenarios representative of the relationship between 2030 emissions and 2100 warming.

Source	Model	Scenario	2100 warming	2030 emissions in $GtCO_2eq$
Ref. [1]	REMIND	Scen135	1.2 °C	25.7
SSP	AIM/CGE	SSP1-26	1.8 °C	37.4
SSP	MESSAGE-GLOBIOM	SSP2-34	2.2 °C	47.1
SSP	AIM/CGE	SSP5-45	2.7 °C	54.0
SSP	IMAGE	SSP2-60	3.3 °C	57.9
SSP	IMAGE	SSP3-Baseline	3.9 °C	62.1
SSP	AIM/CGE	SSP3-Baseline	4.1 °C	66.6
SSP	AIM/CGE	SSP5-Baseline	4.7 °C	67.4
SSP	WITCH-GLOBIOM	SSP5-Baseline	5.1 °C	73.9

3. Supplementary Note

The supplementary data provides:

- the global 2100 median warming assessment of countries' NDCs (shown in Figure 3b)
- the relationship between 2030 national emissions allocations under the CBDR-RC hybrid approach and the underlying global 2100 warming (shown in Figure 3b),
- and the national emissions trajectories until 2100, for all available countries, under the CBDR-RC hybrid approach under the 1.5°C-scenarios and 2°C-scenarios (see article).

This supplementary data can be visualized at: http://paris-equity-check.org/warming-check

In Sheet: {Global 2100-warming of NDCs}

- Column A indicates countries' ISO Alpha-3 codes,
- Column B indicates the full name of countries,
- Column C indicates the Global warming for Average NDC assessment [in °C]
- Column D indicates the Global warming for High NDC assessment [in °C]
- Column E indicates the Global warming for Low NDC assessment [in °C]

In Sheet {2030-allocation vs 2100-warming}

- Row 2 indicates the range of global 2100-warming [in °C] interpolated between the minimum and maximum 2100 warming
- Column A indicates countries' ISO Alpha-3 codes,
- Column B indicates the full name of countries,
- Columns D to OH contain the national 2030 emissions levels associated with the global 2100-warming temperature of Row 1 using the CBDR-RC hybrid approach [in % change compared to 2010 levels]

In Sheets: {CBDR-RC hybrid 1.5°C-scenario}, {CBDR-RC hybrid 1.5°C-scenario %}, {CBDR-RC hybrid 2°C-scenario} and {CBDR-RC hybrid 2°C-scenario %}

- Row 2 indicates the years,
- Column A indicates countries' ISO Alpha-3 codes,
- Column B to IR provides national historical GHG emissions from 1850 to 2010, and CBDR-RC hybrid 1.5°C-scenario and 2°C-scenario allocation for 2011-2100, [in GgCO2eq] and in [% of 2010 levels]

4. Supplementary References

70. Rogelj, J. *et al.* Energy system transformations for limiting end-of-century warming to below 1.5 °C. *Nat. Clim. Chang.* **5,** 519–527 (2015).

71. Baer, P., Fieldman, G., Athanasiou, T. & Kartha, S. Greenhouse Development Rights: towards an equitable framework for global climate policy. *Cambridge Rev. Int. Aff.* **21,** 649–669 (2008).

72. Kemp-Benedict, E. *Calculations for the Greenhouse Development Rights Calculator*. (2010).

73. Meinshausen, M. *et al.* National post-2020 greenhouse gas targets and diversity-aware leadership. *Nat. Clim. Chang.* **5,** 1098–1106 (2015).

74. Meinshausen, M. & Alexander, R. INDC Factsheets. *Australian-German Climate and Energy College* (2015). Available at: http://climatecollege.unimelb.edu.au/indc-factsheets/. (Accessed: 1st January 2015)
