# Supplementary material for: Warming assessment of the bottom-up Paris Agreement emissions pledges
Source: Nat Commun. 2018 Nov 16;9:4810. doi: 10.1038/s41467-018-07223-9 (PMC6240108; doi:10.1038/s41467-018-07223-9)
Supplement: Supplementary file 3 — Description of Additional Supplementary Files [file 41467_2018_7223_MOESM3_ESM.pdf]

Description of Additional Supplementary Files

File Name: Supplementary Data 1
Description: Warming assessment of the bottom-up Paris Agreement emissions pledges
